# Identification and validation of a five-lncRNA prognostic signature related to Glioma using bioinformatics analysis

**DOI:** 10.1186/s12885-021-07972-9

**Published:** 2021-03-09

**Authors:** Chunyu Zhang, Haitao Liu, Pengfei Xu, Yinqiu Tan, Yang Xu, Long Wang, Baohui Liu, Qianxue Chen, Daofeng Tian

**Affiliations:** 1 Department of Neurosurgery, Renmin Hospital of Wuhan University, Wuhan, 430060 Hubei Province People’s Republic of China; 2 Department of Cardiothoracic Surgery, The First Affiliated Hospital of Jiaxing University, Jiaxing, 314001 Zhejiang Province People’s Republic of China; 3 Sun Yat-sen University, The Seventh Affiliated Hospital, Shenzhen, 518000 Guangdong Province People’s Republic of China

**Keywords:** WGCNA, TCGA, CGGA, Glioma, Prognostic signature

## Abstract

**Background:**

To accurately predict the prognosis of glioma patients.

**Methods:**

A total of 541 samples from the TCGA cohort, 181 observations from the CGGA database and 91 samples from our cohort were included in our study. Long non-coding RNAs (LncRNAs) associated with glioma WHO grade were evaluated by weighted gene co-expression network analysis (WGCNA). Five lncRNA features were selected out to construct prognostic signatures based on the Cox regression model.

**Results:**

By weighted gene co-expression network analysis (WGCNA), 14 lncRNAs related to glioma grade were identified. Using univariate and multivariate Cox analysis, five lncRNAs (CYTOR, MIR155HG, LINC00641, AC120036.4 and PWAR6) were selected to develop the prognostic signature. The Kaplan-Meier curve depicted that the patients in high risk group had poor prognosis in all cohorts. The areas under the receiver operating characteristic curve of the signature in predicting the survival of glioma patients at 1, 3, and 5 years were 0.84, 0.92, 0.90 in the CGGA cohort; 0.8, 0.85 and 0.77 in the TCGA set and 0.72, 0.90 and 0.86 in our own cohort. Multivariate Cox analysis demonstrated that the five-lncRNA signature was an independent prognostic indicator in the three sets (CGGA set: HR = 2.002, *p* < 0.001; TCGA set: HR = 1.243, *p* = 0.007; Our cohort: HR = 4.457, *p* = 0.008, respectively). A nomogram including the lncRNAs signature and clinical covariates was constructed and demonstrated high predictive accuracy in predicting 1-, 3- and 5-year survival probability of glioma patients.

**Conclusion:**

We established a five-lncRNA signature as a potentially reliable tool for survival prediction of glioma patients.

**Supplementary Information:**

The online version contains supplementary material available at 10.1186/s12885-021-07972-9.

## Background

Glioma is the most prevalent neoplasm in central nervous system (CNS), with the highest malignancy and the worst prognosis compared with other tumors in brain. In spite of the fact that clinical interventions have been improved dramatically, but the outcomes of glioma patients fail to satisfy the expected goals [1]. The main factors of the poor prognosis are due to the atypical symptoms and the aggressiveness of glioma, resulting in a large proportion of glioma patients diagnosed and treated at the advanced grade [2]. Therefore, further exploration on the molecular mechanisms of tumorigenesis and progression is crucial for improving diagnosis and treatment and investigations into the novel biomarkers are needed.

The past few years witness the great accumulation of evidence indicating the subtle and complex regulatory process in tumor biological behaviors, insights into which in all probability facilitate the development of more effective treatment strategies and improvement in clinical benefits [3]. High-throughput sequencing makes it possible to comprehensively analyze the transcriptome and genome, especially for long non-coding RNA (lncRNA), mRNA, and others [4, 5]. LncRNA typically has more than 200 nucleotides in length, disabling to encode proteins [6]. This type of RNA has been confirmed to be involved in several biological functions including transcription [7], RNA splicing [6], N6-methyladenosine (m6A) [8] and others. The expression profiles of lncRNAs are cell-specific and tumor-specific [9]. Meanwhile, there is mounting evidence that the dysregulated lncRNAs act a pivotal part in many biological processes of malignancy, demonstrating great potentiality as novel diagnostic or prognostic molecular biomarkers [10, 11]. Regarding glioma, some lncRNAs are dysregulated and implicated in cell growth, apoptosis, invasion and angiogenesis. LncRNA-MALAT1 has been considered as a tumor suppressor and down expression of MALAT1 to cause remarkable promotion of invasion and proliferation of the glioma cells [12]. LncRNA-NEAT1 has been revealed to be modulated by the epidermal growth factor receptor (EGFR) pathway, leading to glioblastoma multiform progression by the WNT/β-Catenin Pathway by Scaffolding enhancer of zeste homolog 2 (EZH2) [13]. Nevertheless, the molecular functions and mechanisms of the great majority of lncRNAs remain ill-defined and the research concerning lncRNAs with diagnosis or prognosis potentiality in glioma is still in the initial stage.

In this study, we used the univariate and multivariate Cox regression analysis to develop a prognostic predictor based on multiple lncRNAs for glioma patients. The prognosis prediction accuracy of the signature was evaluated in the CGGA, TCGA and our own cohorts.

## Materials

### Data obtaining

Two sets of data were included in our study. The both lncRNA-sequencing data were downloaded from the TANRIC database [14], meanwhile, the corresponding clinical information were separately obtained from two databases, including the Chinese Glioma Genome Atlas (CGGA, http://www.cgga.org.cn/) and The Cancer Genome Atlas (TCGA, https://portal.gdc.cancer.gov/). The CGGA dataset consisting of 89 LGG and 92 GBM tissue samples, acting as a training set, was used to establish a multi-lncRNA signature. The TCGA dataset, consisting of 405 LGG and 136 GBM observations, and our cohort including 38 LGG and 53 GBM patients were treated as testing and validation sets to examine the statistical significance of the signature. The clinical characteristics of glioma patients in the training, testing and validation sets were shown in Table S1. MRNA expression data of 139 GBM and 181 LGG patients and miRNA microarray data of 101 LGG and 97 GBM samples were downloaded from the CGGA database.

### Weighted gene co-expression network analysis (WGCNA)

The top 35% most variant genes (4455 genes) were subject for WCGNA step by analyzing variance in CGGA set. The process of WGCNA included the construction of gene expression similarity matrix, adjacency matrix, and then co-expression network [15]. The power value of the soft threshold β of the adjacency matrix was set as 6 through the R function pickSoftThreshold. And then we used the ScaleFree plot to evaluate whether the network met the criterion of the scale-free topology network. The hierarchical clustering method on the basis of average-linkage, was adopted to define modules. When clustering, we used the smallest module size of 30. The Pearson correlation coefficients between modules and clinical variables were calculated. The module with absolute value of the correlation coefficient > 0.5 and *p* < 0.01 was worth further analysis.

### Survival analysis

The association between the hub lncRNAs and overall survival (OS) were first calculated using univariate Cox analysis. The lncRNAs with *p* < 0.05 were chosen to carry out multivariate Cox regression analysis and step-wise regression method was used to select variables to construct prognostic model on the basis of the Akaike information criterion (AIC). Then, risk score was obtained based on coefficients (*β*) from multivariate cox analysis and expression data of lncRNAs (*EXPi*). The detailed formula was as follows: risk score = *EXP*1 * *β*1 + *EXP*2 * *β*2 +……+ *EXPi* * *βi*. In the light of the median values of the risk scores in training and validation cohorts, patients with glioma were separated into high and low risk groups, respectively. To analyze whether the survival time of the patients in two risk groups was significantly different, Kaplan-Meier (KM) curve was used and visualized by R package survival. To evaluate the predicting accuracy of the established model, receiver operating characteristic (ROC) curve was conducted.

### Construction of Nomogram survival model

Nomogram could illustrate the relationship between different variables in a graphical way [16]. Meanwhile, the advantage of nomogram is that it can personally calculate the survival rate of specific tumor patients, so it has great value in clinical application [16]. To assess the probabilities of overall survival at 1, 3, and 5 years for glioma patients, a nomogram was constructed here, consisting of risk score, 1p19q status and glioma WHO grading. In addition, calibration curve was used to assess the degree to which actual results were consistent with the predicted results of nomogram. Areas under the ROC curve were used to making a comparison between the predictive reliability of the nomogram and clinical variables.

### Differential expression analysis

LncRNA, miRNA, and mRNA expression data were all log2 transformed using R project (version 4.0.1). We first normalize the log2-transformed data into distribution of mean value and sent which to conduct differential expression between LGG and GBM samples using the Bayesian test from limma package [17]. T-test and Benjamini & Hochberg (BH) method were used to obtain the adjusted *p* value. RNAs with |Log2FC| > 1 and adjusted *p* value< 0.05 were considered expression significantly changed.

### Construction of ceRNA regulatory network and functional annotations

Several lncRNAs have recently been observed to participate in the regulation of gene expression, through the absorption of miRNAs, resulting in the occurrence and development of malignant tumors [18]. Through the starBase v2.0 database [19], we first predicted the hub lncRNA-DEmiRNA interactions. Three online tools, including miRTarBase [20], TargetScan [21] and miRDB [22] were used to explore the targets of the DEmiRNAs and the overlapped genes with differential expression in the three databases were chosen as candidates for ceRNA network. Visualization of ceRNA regulatory network was accomplished by software cytoscape (https://cytoscape.org/). The mRNAs in the ceRNA network were subject to enrichment analysis by the R package clusterProfiler [23] in R project (v4.0.1). Gene Ontology (GO) and Kyoto Encyclopedia of Genes and Genomes (KEGG) pathway enrichment analysis results with *p* < 0.05 were considered significant.

### Tumor specimens

Tumor samples were obtained during operation and stored in liquid nitrogen immediately after isolation before use. The pathological grades were identified according to WHO classification and there were 38 samples classified into WHO II-III glioma and 53 into WHO IV glioma. All methods were performed in accordance with the guideline approved by Ethics Committee of Renmin Hospital of Wuhan University [approval number: 2012LKSZ (010) H].

### Quantitative RT-PCR

Total RNA was extracted from tumor samples using PrimeScriptTM RT Reagent Kit with a gDNA Eraser (Takara Bio Inc., Japan) according to manufacturer protocol and transcribed into cDNA. QRT-PCR was carried out by SYBR Premix Ex Taq (Takara Bio Inc., Japan). The expression levels of lncRNAs were normalized to GAPDH and calculated by the 2^−ΔΔCt^ method. Sequences of the primers and conditions of amplification were shown in Table S2.

## Results

### Co-expression network construction

**Fig. 1:**
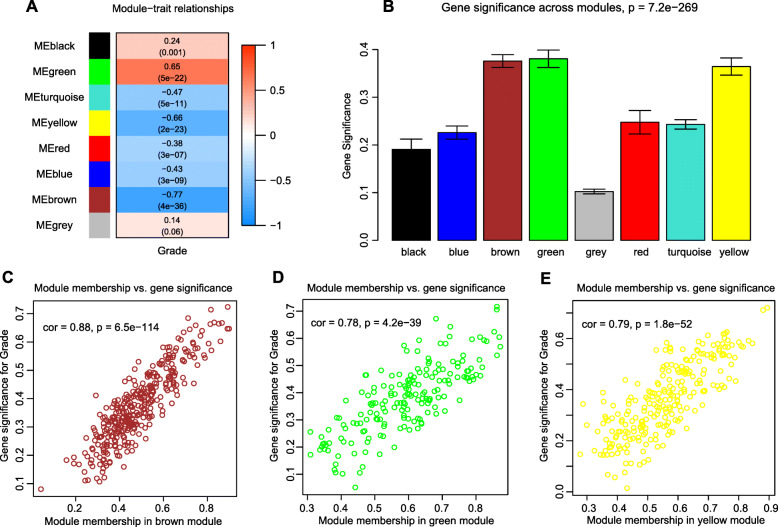
Weighted gene co-expression analysis (WGCNA) of lncRNA expression profiles in the training set. (**a**) Heatmap of Pearson correlation analysis of modules and the clinical trait of glioma. Rows represent module eigengenes and columns traits. (**b**) Barplot of average gene significance in eight modules. The green, yellow and brown modules were worth analyzing. (**c**-**e**) Scatter plot of module eigengenes in brown, green, and yellow modules

Through conjoint analysis of co-expression network and clinical features, modules with biological significance can be confirmed in this step [15]. When the value of *β* was chosen as 6, the scale-free topology fit index reached 0.98, meeting the standard of approximate scale-free topology (Fig. S1). LncRNAs with similar expression patterns were divided into the same module by cluster dendrogram trees and eight modules were obtained here. The results of the investigation into the relationships between modules and glioma WHO grading were presented in Fig. 1a. By setting the threshold values as Pearson correlation coefficient > |0.5| and *p* < 0.01 to select the significant modules, and by inquiring into mean gene significance across all genes in one module, the brown, green and yellow module were considered to be closely related to the WHO grade of glioma (Fig. 1b). Meanwhile, the module membership (MM) vs. gene significance (GS) analysis of the brown, green and yellow modules showed that the three were endowed with higher correlation between MM and GS (Fig. 1c-e). Within these three modules, we obtained 14 hub lncRNAs in total, by selecting lncRNAs with |GS| > 0.85 and |MM| > 0.65.

### Survival analysis of risk score and clinical features

**Fig. 2:**
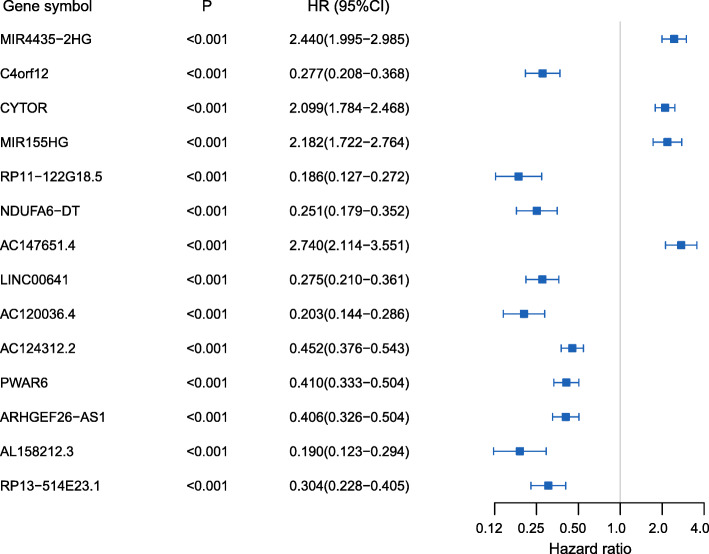
Forest plot of univariate Cox regression analysis of the 14 candidate lncRNAs with overall survival of glioma patients. Abbreviations: HR, hazard ratio; CI, confidence interval

**Fig. 3:**
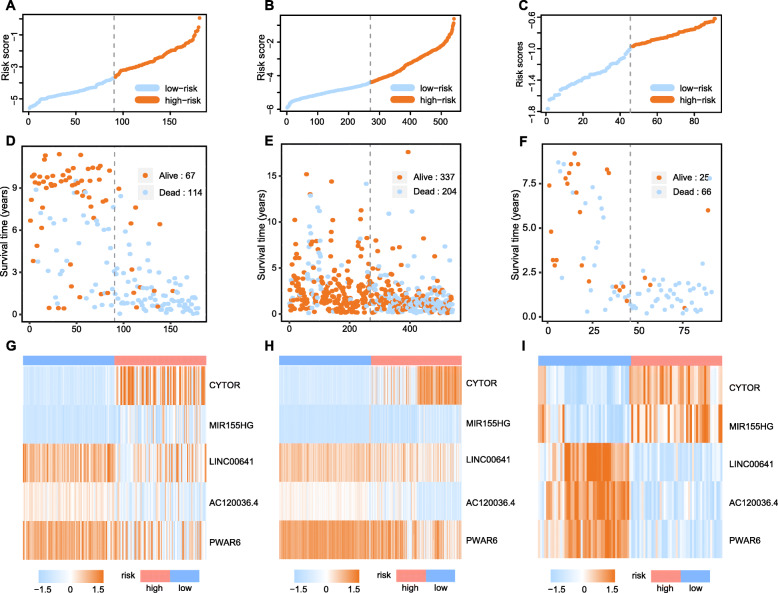
Patients were grouped according to the median risk score in training, testing and validation sets. Scatter plot of distribution of risk scores (**a**-**c**), survival state of glioma patients (**d**-**f**) and heatmap of five hub lncRNAs expression profiles in training, testing and validation (**g**-**i**) cohorts

**Fig. 4:**
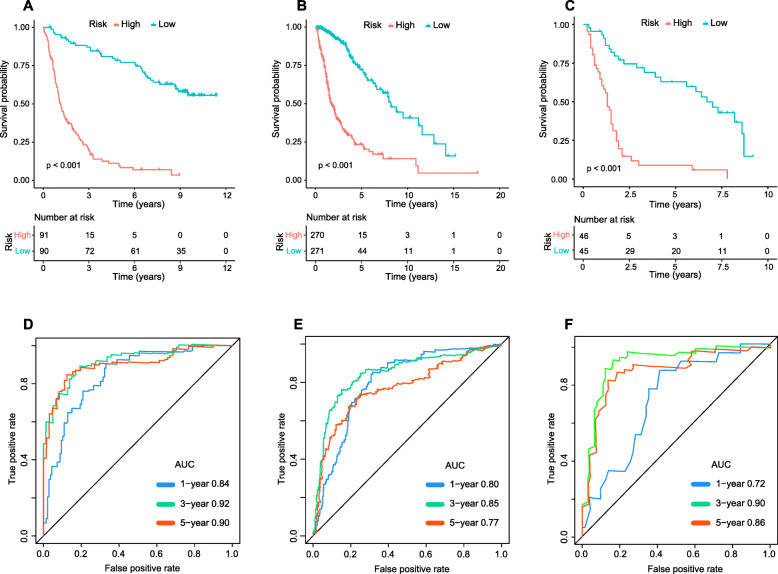
Survival analysis and predictive accuracy assessment of the five-lncRNA signature. Kaplan-Meier curve demonstrated significant survival differences between high and low groups in the training (**a**), testing (**b**) and validation (**c**) sets. Receiver operating characteristic (ROC) curve showed the great predictive accuracy of the signature in the training (**d**), testing (**e**) and validation (**f**) sets

Survival analysis had been firstly performed on the training set of 181 glioma patients. From the univariate Cox analysis, 14 hub lncRNAs detected in the step of WGCNA were considered to be statistically connected with the clinical outcomes of gliomas (Fig. 2), and 5 of which were selected to establish prognostic model based on step-wise Cox analysis (Table S3). Subsequently, the risk score system based on the step regression Cox analysis was constructed, and the following formula was adopted here: risk score = CYTOR * 0.3447 + MIR155HG * -0.8509 + LINC00641 * -0.7135 + AC120036.4 * -0.5505 + PWAR6 * -0.5748. The median risk scores of samples was calculated separately in the training, testing and validation sets, and set as cutoffs in risk stratification for glioma patients (Fig. 3a-f). And the expression profiles of the five lncRNAs were visualized by heat map in the training (Fig. 3g), testing (Fig. 3h) and validation (Fig. 3i) datasets. Moreover, we used the K-M curve evaluated whether there were significant survival time differences for glioma patients after risk stratification. The results showed the mortality of patients in the high-risk group was significantly higher than that in the low-risk group (Fig. 4a-c). The values of AUC of the signature at 1-, 3-, and 5-year OS were 0.84, 0.92 and 0.90 in the training set; 0.8, 0.85 and 0.77 in the testing cohort and 0.72, 0.90 and 0.86 in validation cohort, demonstrating the great reliability of the prognosis signature (Fig. 4d-f). Univariate Cox regression analysis suggested that risk score, 1p19q status and WHO grading had prognostic values (*p* < 0.05) in the three sets, while age and sex not. Then, in multivariate Cox regression analysis of risk score and clinicopathological risk variables, risk score was still an independent and powerful prognosis-predicting factor (training set: HR = 2.002, 95%CI [1.584–2.530], *p* < 0.001; testing set: HR = 1.243, 95%CI [1.063–1.469], *p* = 0.007; validation set: HR = 4.457, 95%CI [1.472–13.491], *p* = 0.008) (Table 1).

**Table 1:** Univariate and multivariate Cox regression analysis of the risk score, clinical variables, and survival in the training, testing and validation cohorts

Variates	Univariate Cox analysis			Multivariate Cox analysis		
	HR	95%CI	P	HR	95%CI	P
**CGGA glioma training set (*n*** **= 181)**						
Grade (ref. WHO IV)	0.101	0.070–0.171	**< 0.001**	0.377	0.208–0.682	**0.001**
Sex (ref. Female)	0.966	0.664–1.404	0.854			
Age (median years, ref. < 42)	1.91	1.310–2.786	**< 0.001**	0.937	0.633–1.387	0.746
1p19q status (ref. codeletion)	5.442	2.901–10.206	**< 0.001**	2.6	1.337–5.048	**0.005**
Risk score (continuous)	2.718	2.269–3.256	**< 0.001**	2.002	1.584–2.530	**< 0.001**
**TCGA glioma testing set (*n*** **= 541)**						
Grade (ref. WHO IV)	0.106	0.079–0.143	**< 0.001**	0.327	0.204–0.500	**< 0.001**
Sex (ref. Female)	1.129	0.854–1.495	0.394			
Age (median, ref. < 42)	5.25	3.654–7.544	**< 0.001**	3.954	2.005–4.163	**< 0.001**
1p19q status (ref. codeletion)	4.793	2.951–7.785	**< 0.001**	3.166	1.873–5.352	**< 0.001**
Risk score (continuous)	2.076	1.853–2.325	**< 0.001**	1.243	1.063–1.469	**0.007**
**Our glioma validation set (*n*** **= 91)**						
Grade (ref. WHO IV)	0.062	0.028–0.136	**< 0.001**	0.057	0.021–0.155	**< 0.001**
Sex (ref. Female)	0.685	0.419–1.12	0.131			
Age (median, ref. < 42)	1.026	0.628–1.677	0.917			
1p19q status (ref. codeletion)	2.033	1.002–4.125	**0.049**	2.758	1.275–5.969	**0.009**
Risk score (continuous)	18.376	6.881–49.075	**< 0.001**	4.457	1.472–13.491	**0.008**

Abbreviations: *HR* hazard ratio; *CI* confidence interval

### Nomogram construction and accuracy assessment

**Fig. 5:**
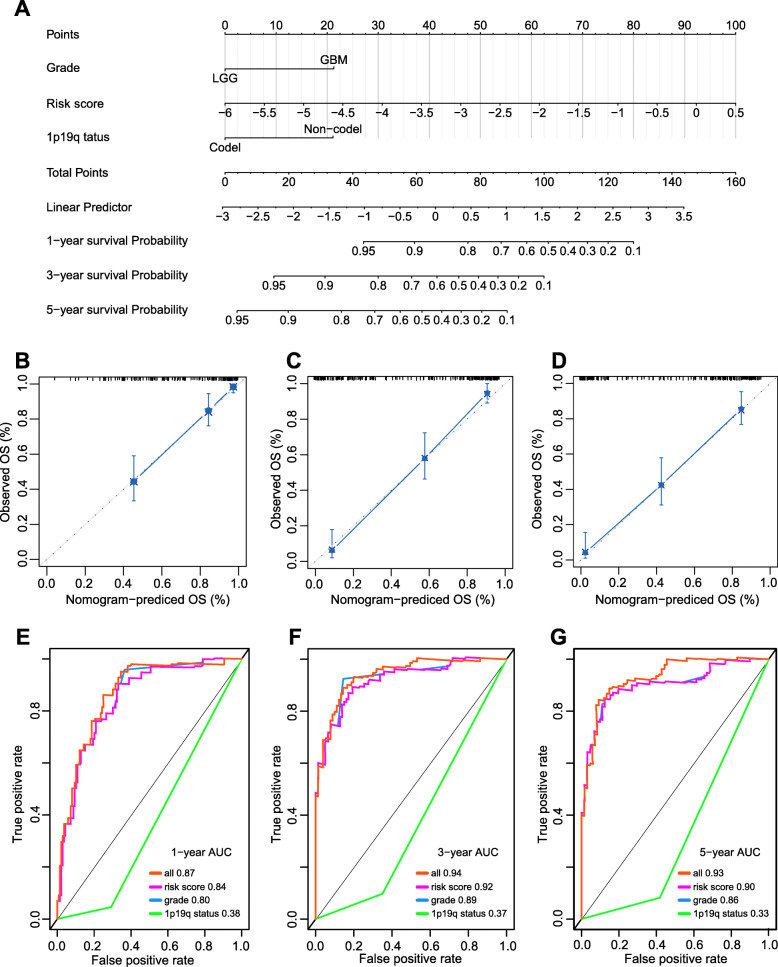
Nomogram construction. A nomogram predicted the survival possibility of gliomas in the training set (**a**). Calibration plot for the nomogram demonstrated the observed and predicted OS probabilities were highly consistent (**b**-**d**). ROC curve assessed the prognosis prediction accuracy of the nomogram at 1-, 3- and 5-year overall survival (**e**-**g**). Abbreviations: OS, overall survival; AUC, area under curve

To facilitate the clinical prognosis assessment for glioma patients, we established a nomogram to perform the prediction of the overall survival probability at 1-, 3-, and 5-year in the training, testing, validation cohorts (Fig. 5a and Fig. S2A-B). The observed and predicted probabilities for the specific observations decrease along the diagonal line in calibration plot (Fig. 5b-d). To measure the predictive accuracy of the merged nomogram and the clinical risk factors at 1-, 3- and 5-year OS, the values of the area under the ROC curve were calculated. The 1-year AUC was 0.87 for nomogram, 0.80 for glioma WHO grading, 0.38 for 1p19q status (Fig. 5e). In assessing the predicting efficacy at 3-year OS, AUC was 0.94 for nomograph, 0.89 for glioma WHO grading, 0.37 for 1p19q status (Fig. 5f). Furthermore, the 5-year AUC was 0.93 for alignment chart, 0.86 for glioma WHO grading, and 0.33 for 1p19q status (Fig. 5g).

### Differential expression analysis of RNAs

We confirmed that the 5 hub lncRNAs were expression-dysregulated between LGG and GBM tissues. In comparison with expression levels of lncRNAs in low grade glioma, CYTOR and MIR155HG were significantly over-expressed in GBM tissues, while three lncRNAs (LINC00641, AC120036.4, PWAR6) were remarkably down-regulated in GBM samples (Fig. S3A-C). Meanwhile, there were 1801 mRNAs and 93 miRNAs upregulated; and 1287 mRNAs and 91 miRNAs downregulated in GBM samples (Fig. S3D, E).

### Construction of ceRNA regulation network and enrichment analysis

**Fig. 6:**
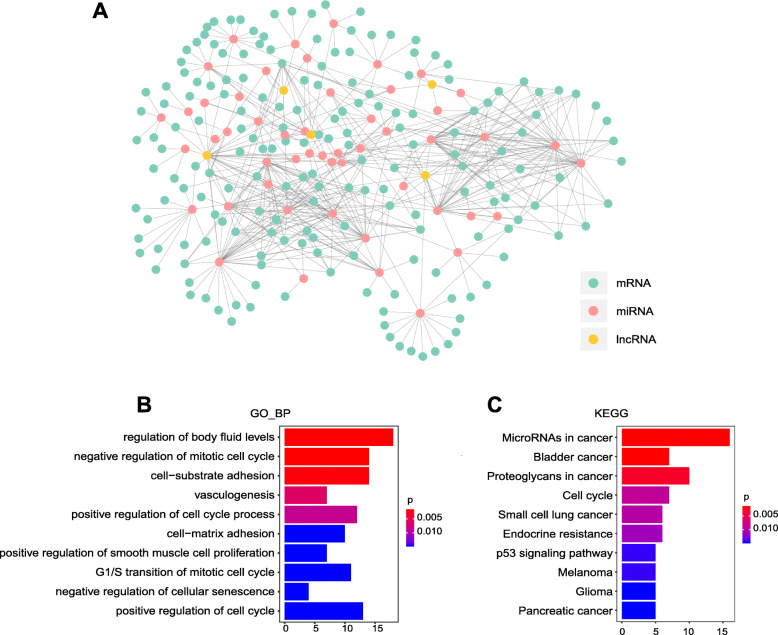
The ceRNA network construction and function annotation. The ceRNA regulatory network contained 61 miRNAs, 182 mRNAs, and 5 lncRNAs (**a**). Nodes represented RNAs and lines interactions between RNAs. GO enrichment analysis of mRNAs in ceRNA network (**b**). Pathway enrichment analysis of mRNAs in ceRNA network (**c**). Abbreviations: ceRNA, competing endogenous RNA; GO, gene ontology; BP, biological process

A competing endogenous RNA (ceRNA) regulation network was constructed and visualized by Cytoscape software (Fig. 6a). And in the network, there were 61 miRNAs, 182 mRNAs, 5 lncRNAs, and 512 edges. Moreover, we explored the possible biological mechanisms of the hub lncRNAs related to glioma. By applying R package clusterProfiler, mRNAs in the network were subject to function annotation analysis. It indicated that the mRNAs regulated by the hub lncRNAs were mainly involved in regulation of cell cycle process, regulation of cellular senescence and in cell−matrix adhesion. Moreover, pathways analysis of target mRNAs showed a statistically significant association with glioma and p53 signaling pathway, confirming potential roles in cancer development of the hub lncRNAs (Fig. 6b, c).

## Discussion

Recent research demonstrates multiple biomarkers integrated into a single model can greatly improve the prognostic value of a single model [24]. Glioma patients are rather heterogeneous. Even in the same pathological grade, there are considerable differences in their clinical outcomes [1]. The present study was designed to detect and validate a signature containing multiple markers to improve the prognostic accuracy for gliomas.

In particular, we applied WGCNA to glioma patients with RNA-seq lncRNA expression data, which has been extensively utilized in exploring biomarkers in cancers such as pancreatic carcinoma and breast cancer [25, 26]. In our research, through the construction of a lncRNA co-expression network with data available at the TANRIC database, three modules-green, yellow and brown associated with glioma WHO grading were identified using the WGCNA algorithm. Total 14 hub lncRNAs with |GS| > 0.85 and |MM| > 0.65 were selected from the three modules. Then, univariate Cox analysis of the hub lncRNAs and overall survival was firstly conducted and demonstrated all the variates having outstanding statistical significance. In multivariate Cox analysis, stepwise regression was used to select markers and a risk model to was constructed to predict glioma prognosis. Finally, five lncRNAs (CYTOR, MIR155HG, LINC00641, AC120036.4 and PWAR6) were chosen to establish a risk score system for prediction the prognosis of gliomas. The signature remained a strong and independent prognostic indicator for OS in the training, testing and validation cohort by inclusion of statistically significant clinical factors determined by univariate Cox regression analysis and the risk signature into multivariate Cox regression analysis. In assessing of model predictive performance, the 1-year, 3-year, and 5-year AUC values in the training and validation cohort were all more than 0.75. Several previous studies associated with construction of prognostic model for glioma patients provided the AUC values of the model. Wang et al. established prognostic model for glioma, and the AUC values in predicting 1-year survival for glioma were TCGA: 0.623 and CGGA: 0.607; and 3-year survival: TCGA: 0.735 and CGGA: 0.803 [27]. Lin et al. used four genes (TAGLN2, PDPN, TIMP1, EMP3) to build prognostic model for glioma and its AUC values were 0.80 in TCGA cohort and 0.72 in CGGA cohort [28].

The results indicated that our predictor has higher accuracy in predicting the prognosis of glioma patients at three prediction time points. Nomogram has been widely applied in cancer prognosis assessment for providing an opportunity of predicting individual survival probability. Here, we constructed a robust nomogram composed of risk score, grade and 1p19q status to predict the prognosis of glioma patients. In addition, the AUC values of the integrated nomogram at 1 year, 3 years and 5 years were greater than the AUC values of grade, 1p19q status and the five-lncRNA signature. The calibration curves demonstrated that the predicted and observed probabilities shown great consistency with each other. These results revealed that the nomogram performed well in predicting the prognosis of glioma patients.

In the TCGA, CGGA and Our datasets, differential expression analysis showed that CYTOR and MIR155HG were significantly overexpressed in GBM compared to LGG samples, while AC120036.4, LINC00641 and PWAR6 showed an inverse expression pattern in glioma samples. Insights into the molecular function of lncRNAs in glioma would accelerate their clinical research and practice. Among the hub lncRNAs, some have been proven to be related to glioma. Recent research reveals overexpression of CYTOR in glioma tissues correlated with metastasis and knockdown of CYTOR can attenuate the tumor cell proliferation and invasion [29]. MIR155HG has been considered to be significantly upregulated in GBM samples in comparison with LGG tissues [30]. The expression of MIR155HG has been found to be positively correlated with tumor grade and prognosis of glioma patients. Meanwhile, MIR155HG knockdown decreases glioma cell proliferation both in vitro and in vivo [30]. Recent study demonstrates MIR155HG is highly expressed in mesenchymal GBM tissues and suppression of MIR155HG can decrease expression of mesenchymal transition-associated proteins, such as β-catenin, N-cadherin, suggesting a function as a regulator in mesenchymal transition progression [31]. Identical to our research, recent bioinformatics analysis reveals PWAR6 and LINC00641 are expression-dysregulated in glioma tissues [32–34]. Meanwhile, PWAR6 has also been considered as a modulator of tumor immunoreaction and epithelial-mesenchymal transition (EMT) [32]. For LINC00641 in glioma, recent research suggested LINC00641 act as an inhibitor of glioma cell proliferation by targeting miR-4262/NRGN axis [35]. However, the in-depth analysis of mechanisms of AC120036.4 has not been identified in glioma.

A huge number of putative lncRNAs have been predicted or validated in human. However, the functions or molecular mechanisms of the great part of lncRNAs remain unclear. To infer the potential roles of the hub lncRNAs with dysregulated expression levels, we constructed a lncRNA-miRNA-mRNA regulatory network. We found that several miRNAs in our ceRNA regulatory network are mainly involved in glioma pathway. Namely miR-155 and miR-107 are associated with poor prognosis in glioma patients and involved in cell proliferation by changing the tumor cell cycle [36, 37]. And the target genes such as WEE1 (WEE1 G2 checkpoint kinase) and CDK6 (cyclin-dependent kinase 6) in our ceRNA network have been considered as key regulators in cell cycle and proliferation [38, 39]. Meanwhile, based on the mRNAs in the network, we carried out the enrichment analysis. Pathway enrichment analysis indicated that the genes were mainly enriched in p53 signaling pathway, which play a sophisticated role in the progression of glioma [40], demonstrating the hub lncRNAs were expected to be involved in the glioma biogenesis and development.

## Conclusion

In summary, the current study utilized comprehensive bioinformatics analysis to determine a risk signature based on lncRNAs, providing a potential tool for assessment of clinical prognosis of patients with glioma.

## Supplementary Information

**Additional file 1 Table S1**. Demographics and clinical characteristics of glioma patients in the training, testing and validation cohorts.

**Additional file 2 Table S2**. Primers for qRT-PCR.

**Additional file 3 Table S3**. The results of multivariate Cox analysis in the training set.

**Additional file 4 Fig. S1**. As shown in the plots, the approximate scale-free topology was obtained when the soft threshold power was about 6.

**Additional file 5 Fig. S2**. A nomogram predicted the survival possibility of gliomas in the TCGA testing (A) and Our validation (B) cohorts.

**Additional file 6 Fig. S3**. Differential expression analysis of the five hub lncRNAs in the CGGA (A) and TCGA (B) and our cohorts (C). Differential expression analysis of mRNAs (D) and miRNAs (E) in CGGA set. (n.s, *p* > 0.05; *, *p* < 0.05; **, *p* < 0.01; and ***, *p* < 0.001).

## Data Availability

The data that support the findings of this study are available in TCGA database at https://portal.gdc.cancer.gov/, at TANRIC database https://bioinformatics.mdanderson.org/public-datasets/, and at CGGA database http://www.cgga.org.cn/.

## Abbreviations

LGG: low grade glioma
GBM: glioblastoma multiforme
lncRNAs: long non-coding RNAs
CGGA: Chinese Glioma Genome Atlas
TCGA: The Cancer Genome Atlas
TANRIC: The Atlas of Noncoding RNAs in Cancer
ROC: calibration
AUC: area under curve
WGCNA: Weighted gene co-expression network analysis
OS: overall survival
HR: hazard ratio
CI: confidence interval
DEmRNA: differential expressed mRNA
DEmiRNA: differential expressed miRNA
GO: Gene Ontology
KEGG: Kyoto Encyclopedia of Genes and Genomes
ceRNA: competing endogenous RNA
EMT: epithelial-mesenchymal transition
