# Supplementary material for: Identification and validation of a five-lncRNA prognostic signature related to Glioma using bioinformatics analysis
Source: BMC Cancer. 2021 Mar 9;21:251. doi: 10.1186/s12885-021-07972-9 (PMC7941710; doi:10.1186/s12885-021-07972-9)
Supplement: Supplementary file 1 — Additional file 1 Table S1. Demographics and clinical characteristics of glioma patients in the training, testing and validation cohorts. [file 12885_2021_7972_MOESM1_ESM.docx]

| **Table S1. Demographics and clinical characteristics of glioma patients in the training, testing and validation cohorts.** | | | | | | |
| --- | --- | --- | --- | --- | --- | --- |
| **Variables** |  | **Training Cohort (n=181)** |  | **Testing Cohort (n=541)** |  | **Validation Cohort (n=91)** |
|  |  | n |  | n |  | n |
| **Age (median, years)** |  |  |  |  |  |  |
| <42 |  | 95 |  | 215 |  | 42 |
| ≥42 |  | 86 |  | 326 |  | 49 |
| **Sex** |  |  |  |  |  |  |
| Female |  | 72 |  | 230 |  | 36 |
| Male |  | 109 |  | 311 |  | 55 |
| **Grade** |  |  |  |  |  |  |
| LGG |  | 89 |  | 405 |  | 38 |
| GBM |  | 92 |  | 136 |  | 53 |
| **1p19q status** |  |  |  |  |  |  |
| Codeletion |  | 42 |  | 138 |  | 17 |
| Non-codeletion |  | 139 |  | 403 |  | 74 |
