# Supplementary material for: Identification and validation of a five-lncRNA prognostic signature related to Glioma using bioinformatics analysis
Source: BMC Cancer. 2021 Mar 9;21:251. doi: 10.1186/s12885-021-07972-9 (PMC7941710; doi:10.1186/s12885-021-07972-9)
Supplement: Supplementary file 2 — Additional file 2 Table S2. Primers for qRT-PCR. [file 12885_2021_7972_MOESM2_ESM.docx]

| Table S2, Primers for qRT-PCR. | |
| --- | --- |
| Gene | Sequences（5'-3') |
| CYTOR-F | AAAATCACGACTCAGCCCCC |
| CYTOR-R | AATGGGAAACCGACCAGACC |
|  |  |
| MIR155HG-F | TTCAAGAACAACCTACCAGAGACCT |
| MIR155HG-R | CTACAGCAAGCCTTCAGCACTC |
|  |  |
| PWAR6-F | CCAGTAGCATAACTCTCTCTCCTCC |
| PWAR6-R | CCAGACTCAAAGTAACCCAACC |
|  |  |
| LINC00641-F | CAGGCCGATAGGCTGTCAAA |
| LINC00641-R | GTGGTCCCTGCTCTTGAGTT |
|  |  |
| AC120036.4-F | TTGATGCCTCTCTGTCACCCAC |
| AC120036.4-R | CAGTTCTCCCTTTGCCATTGT |
|  |  |
| GAPDH-F | CCTCGTCCCGTAGACAAAATG |
| GAPDH-R | TGAGGTCAATGAAGGGGTCGT |
| F: Forward; R: Reverse; conditions of amplification: at 95°C for 30 s, then 40 cycles of amplification at 95°C for 5 s, at 59°C for 30 s afterward, finally at 72°C for 30 s. | |
