# Supplementary material for: Identification and validation of a five-lncRNA prognostic signature related to Glioma using bioinformatics analysis
Source: BMC Cancer. 2021 Mar 9;21:251. doi: 10.1186/s12885-021-07972-9 (PMC7941710; doi:10.1186/s12885-021-07972-9)
Supplement: Supplementary file 3 — Additional file 3 Table S3. The results of multivariate Cox analysis in the training set. [file 12885_2021_7972_MOESM3_ESM.docx]

| Table S3. The results of multivariate Cox analysis in the CGGA set | | | | | | |
| --- | --- | --- | --- | --- | --- | --- |
| **lncRNA** | **coef** | **exp(coef)** | **se(coef)** | **z** | **Pr(>\|z\|)** | **95%CI** |
| CYTOR | 0.3447 | 1.4115 | 0.1781 | 1.935 | 0.053025 | 0.9955-2.0014 |
| MIR155HG | -0.8509 | 0.427 | 0.229 | -3.716 | 0.000203 | 0.2726-0.6689 |
| LINC00641 | -0.7135 | 0.4899 | 0.2006 | -3.557 | 0.000375 | 0.3307-0.7259 |
| AC120036.4 | -0.5505 | 0.5766 | 0.2827 | -1.948 | 0.051475 | 0.3313-1.0035 |
| PWAR6 | -0.5748 | 0.5628 | 0.1645 | -3.494 | 0.000475 | 0.4077-0.7769 |

Abbreviations: Coef: coefficient; CI: confidential interval.
